# Properties and Mechanisms of TBBPA and TBBPS Adsorption onto Various Soils in China

**DOI:** 10.3390/toxics13080686

**Published:** 2025-08-18

**Authors:** Qi Wang, Aiguo Gu, Hongzhen Lian, Jie Zou

**Affiliations:** 1 Jiangsu Product Quality Testing and Inspection Institute, Nanjing 210007, China; qi.wang@mail.ecust.edu.cn (Q.W.); 18061777400@163.com (A.G.); 2 State Key Laboratory of Analytical Chemistry for Life Science, School of Chemistry and Chemical Engineering, Nanjing University, Nanjing 210023, China

**Keywords:** tetrabromobisphenol A, tetrabromobisphenol S, soil type, adsorption, humin, huminic acid

## Abstract

Understanding the differences in the adsorption behaviors of tetrabromobisphenol A (TBBPA) and tetrabromobisphenol S (TBBPS) on soils is critical for assessing their environmental mobility and risks. This study investigated the adsorption characteristics and patterns of TBBPA/S across various soil types. Adsorption kinetics analysis indicated that the adsorption of TBBPA/S on soils followed pseudo-secondary-order kinetics. Isotherm results revealed that the Langmuir model described TBBPA adsorption more accurately, while the Freundlich model was a better fit for TBBPS adsorption, suggesting distinct adsorption mechanisms due to their differing properties. Correlation analysis and principal component analysis (PCA) were performed to identify the key soil physicochemical properties influencing TBBPA/S adsorption. The results showed that TBBPA adsorption was inversely correlated with soil pH and positively correlated with clay content. In contrast, TBBPS adsorption displayed negative correlations with soil pH and sand content, and positive correlations with amorphous iron, amorphous aluminum, and free iron content. Further analysis of different treated soil fractions demonstrated that soil organic matter dominated the adsorption of TBBPA/S, with humic acid playing a more significant role than humin. The adsorption behavior characteristics of TBBPA/S on different soils provide fundamental data for understanding their environmental fate in soil systems.

## 1. Introduction

Brominated flame retardants (BFRs) are a class of organic compounds used to suppress combustion. BFRs are commonly used as additives or raw materials in the production of plastics, electronics, construction and textiles, and other industrial products [1]. Tetrabromobisphenol A (TBBPA) is one of the most widely used BFRs globally, accounting for approximately 60% of the total BFR production [2]. The International Agency for Research on Cancer (IARC) of the World Health Organization has classified TBBPA as a Class 2A carcinogen. Consequently, tetrabromobisphenol S (TBBPS) has increasingly been adopted as a substitute for TBBPA [3,4,5]. TBBPA was proved to function as an endocrine disruptor and induce oxidative stress [6], apoptosis, neurotoxicity [7], and immunotoxicity [8]. Although few studies exist on the toxicity of TBBPS, some have still found evidence of neurodevelopmental and hepatotoxic [9,10,11,12]. Therefore, understanding the environmental fate of TBBPA/S is critical for assessing their ecotoxicity.

TBBPA/S have a high octanol–water partition coefficient, indicating their tendency to accumulate in soils. Studies havse shown that the concentration of TBBPA/S in surface water systems, such as rivers, lakes, and estuaries, are significantly lower than those found in soils systems [13,14]. At open-air incineration site and informal electronic dismantling sites in Qingyuan, Guangdong Province, China, TBBPA levels commonly exceed 20 ng g^−1^ dry weight (dw), with some reaching as high as 646.04 ng g^−1^ dw [15]. In industrial parks in Shandong, China, TBBPA/S and their derivatives have also been detected [1]. Therefore, the environmental fate of TBBPA/S in soils should be given more attention. Due to the complex chemical composition of soils, different regions exhibit varying adsorption capacities of TBBPA/S, and there is a lack of comprehensive data on the adsorption process of TBBPA/S in different types of soils. Critically, the adsorption mechanisms of TBBPA/S across diverse soil types remain poorly characterized. A few studies have explored TBBPA adsorption onto solid phases, such as sediments [16], biochar [17], and individual soil [18], but a systematic analysis of TBBPA and TBBPS adsorption behaviors across various soil types has not been conducted. While soil organic matter (SOM) and pH are recognized as key adsorption determinants [16,18], mineralogical influences (e.g., ferromanganese oxides) remain overlooked. Therefore, it is essential to evaluate the effects of SOM (e.g., humin and humic acid), minerals, and soil pH on the adsorption of TBBPA/S onto different soils.

This study quantifies adsorption kinetics and isotherms of TBBPA/S across heterogeneous soils to resolve these mechanisms. The effects of SOM (humin and humic acid), pH, particle composition, cation exchange capacity (CEC), and ferromanganese oxides were assessed. In addition to correlation analysis, principal component analysis (PCA) was conducted to identify dominant factors influencing adsorption, utilizing soil adsorption coefficients (K_d_) and soil properties.

## 2. Materials and Methods

### 2.1. Chemical Reagents

Tetrabromobisphenol A (C_15_H_12_Br_4_O_2_, TBBPA) and Tetrabromobisphenol S (C_12_H_6_Br_4_O_4_S, TBBPS) were obtained from AccuStandard (New Haven, CT, USA). Sodium hydroxide (GR, NaOH), calcium chloride (GR, CaCl_2_), and methanol [MeOH high-performance liquid-chromatography-grade (HPLC)] were obtained from Aladdin Biochemical Technology Co.,Ltd., Shanghai, China. Hydrogen peroxide (30%, H_2_O_2_), hydrochloric acid (GR, HCl), and hydrofluoric acid (GR, HF) were obtained from Sinopharm chemical reagent Co., Ltd., Shanghai, China. All solute ions were prepared with ultrapure water using a Milli-Q System. TBBPA is a brominated derivative of bisphenol A (Figure S1a), with a molecular weight of 543.87. It is a white powder at room temperature, with a density of 2.12 g/cm^3^, a melting point of 179–184 °C, a boiling point of 316 °C, and a solubility in water of 4.15 mg/L at 25 °C. It is soluble in ethanol, ether, benzene, and chloroform. The octanol water partition coefficient logKow (25 °C) is 6.53, vapor pressure is 4.72 × 10^−9^ Pa, Henry’s law constant is 1.47 × 10^−5^ Pa/(mol·m^3^), and the dissociation constants pKa_1_ are 7.50, and pKa_2_ are 8.50 [19]. TBBPS is a brominated derivative of bisphenol S (Figure S1b), with a molecular weight of 565.85. It is a white powder at room temperature, with a density of 2.36 g/cm^3^, a melting point of ≥280 °C, a boiling point of 539.4 °C, and slightly solubility in water. It is soluble in methanol, ethanol, and chloroform. The octanol water partition coefficient logKow (25 °C) is 5.2; the dissociation constants are as follows: pKa_1_ is 4.74, and pKa_2_ is 5.35.

### 2.2. Soil Types

Eight representative soil samples were collected from different regions of China for this study. These included black soil from Helen, Heilongjiang (HLG), fluvo-aquic soils from Liaocheng, Shandong (SD), chestnut soil from Ningxia (NX), red soil from Jiangmen, Guangdong (GD), limestone soil from Chongqing (CQ), dark brown forest soil from Suining, Sichuan (SC), paddy soil from Suzhou, Jiangsu (JS), and red soil from Yingtan, Jiangxi (JX). The specific physicochemical properties of the tested soils are shown in Table S1. The tested soils were air-dried, large stones and plant roots were removed, and the samples were then crushed and sieved through a 1 mm sieve to obtain clean soil. Neither TBBPA nor TBBPS were detected in all tested soil samples.

### 2.3. Determination and Characterization of Selected Physicochemical Properties of Soil

According to Supplementary File Text S1, the physicochemical properties of the soils were determined using Chinese standard methods.

### 2.4. Preparation of Soil Components

To investigate the effect of soil components on TBBPA/S adsorption, samples of different components were prepared from clean soil.

#### 2.4.1. Oxidized Soil (H_2_O_2_ Treatment)

Overall, 30% of H_2_O_2_ solution was added to the tested soil samples in small quantities several times until no more bubbles were produced after the addition of the H_2_O_2_ solution, and then the samples were air-dried.

#### 2.4.2. Removal of Organic Matter from Soil (Calcination)

The tested soil samples were put into the crucible with a lid, and placed in a muffle furnace at 110 °C for 6 h, followed by calcination at 400 °C for 12 h. They were then taken out of the crucible after cooling to room temperature.

#### 2.4.3. Soil Extraction of Humic Acid

Humic acid and humin were extracted from soil according to the method of International Humic Substances Society (IHSS). Then, 100 g of clean soil was placed in a 2000 mL glass beaker, and the pH was adjusted to 2.0 using 1 M HCl, followed by the addition of 1000 mL of 0.1 M HCl. After shaking for 30 min, the mixture was centrifuged to separate the soil from the liquid. The above operations were repeated three times. The remaining soil was collected, and the pH was adjusted to 7.0 with 2 M NaOH. Subsequently, 1000 mL of 0.1 M NaOH was added, and after 30 min of shaking, the mixture was centrifuged to separate the soil from the liquid. The process was repeated until the solution turned colorless or pale yellow. The extracted solution was the crude humic acid, and the precipitate was the crude humin. The pH of the crude humic acid was adjusted to 2.0 using 6 M HCl, and 20 g of anhydrous sodium sulfate was added. After standing for 12 h, the mixture was centrifuged to separate the precipitate from the liquid. The above operations were repeated until the solution was colorless or light yellow. The precipitate was collected and dissolved in a small amount of 0.1 M NaOH solution, and centrifuged to remove impurities. A mixture of 0.1 M HCl: 0.3 M HF (1:1) was then added into the solution, and the precipitate was collected by centrifugation. This step was repeated three times to obtain humic acid. Humic acid was dissolved in a small amount of 0.1 M NaOH in a dialysis bag (3500–5000 Da) and dialyzed with ultrapure water. The water was changed every 4 h until no Cl^−^ could be detected in the external solution. Finally, the precipitate was collected and freeze-dried to obtain purified humic acid.

#### 2.4.4. Soil Extraction of Humin (Humin)

The abovementioned crude humin was placed in a 2000 mL beaker, and we added 1000 mL of 1 M HF and 2 M HCl (volume ratio of 1:1). The beaker was then placed in a water bath at 80 °C for 4 h. The mixture was centrifuged and we discarded the supernatant after shaking for 30 min. This procedure was repeated three times. The remaining solid was washed with ultrapure water until no Cl^−^ could be detected in the washing solution. The precipitate was then collected and freeze-dried to obtain purified humin.

### 2.5. Experimental Procedure

#### 2.5.1. Adsorption Kinetic Experiments

Then 0.01 M of NaOH solution was mixed with a specific amount of TBBPA or TBBPS to prepare a 100 mg/L TBBPA alkaline bulk solution and 250 mg/L TBBPS alkaline bulk solution. The bulk solution was diluted to 2 mg/L TBBPA and 5 mg/L TBBPS, respectively, and a certain amount of CaCl_2_ was added to give a concentration of 0.01 M. Subsequently, 10 mL of the mixture was dispensed into 50 mL centrifuge tubes, and 0.5 g of clean soil was added to each centrifuge tube. The centrifuge tubes were placed in a dark 25 °C constant-temperature shaker and shaken at 200 rpm for 48 h. At specified time intervals (0.25, 0.5, 1, 4, 8, 12, 24, 48 h), the tubes were drawn out and centrifuged for 5 min at 2100× *g*. The supernatant was filtered through a 0.45 μm glass-fiber membrane, and the filtrate was analyzed for TBBPA/S concentrations using high-performance liquid chromatography (HPLC). Three repetitions were set up for each treatment. Previous experiments showed that the recoveries of TBBPA and TBBPS in soils during the experiment were 82.4 ± 6.8% and 96.5 ± 3.6%, respectively.

#### 2.5.2. Adsorption Isotherm Experiments

Then, 0.5 g of clean soil or 0.1 g of soil component was mixed with 0.01 M CaCl_2_ solutionin with the water–soil ratio at 20:1, and we added a certain amount of TBBPA and TBBPS (0, 0.5, 0.75, 1.0, 1.5, 2.0, 2.5 mg/L). The centrifuge tubes were placed in a dark 25 °C constant-temperature shaker and shaken at 200 rpm for 24 h; then, the TBBPA and TBBPS concentrations were determined. In order to avoid the influence of pH, the solution pH was adjusted to 7.0 during the adsorption isotherm experiments involving soil components.

All adsorption kinetics and isotherm experimental data were obtained through three repeated experiments. Blank samples were set up for each experiment to eliminate errors in the experimental process.

### 2.6. Analytical Methods

The concentration of TBBPA/S in the soil and solution was analyzed according to Supplementary File Text S2. The detection limit of the analytical method used in this study for TBBPA was 3 μg/L, and the detection limit for TBBPS was 10 μg/L.

### 2.7. Mathematical Model

#### 2.7.1. Adsorption Kinetic Modeling

##### The Pseudo-First-Order Kinetic Model

The pseudo-first-order kinetic model, in which the adsorption rate is linearly related to the concentration of the target pollutant, has been widely applied to adsorption processes in solid–liquid systems [20]. The equation is expressed as follows:

(1) $$Q_{t}\;=\;Q_{e}(1-e^{-k_{1}t})$$

where Q_t_ is the concentration of adsorbed pollutant at time t (mg kg^−1^), Q_e_ is the concentration of adsorbed pollutant at adsorption equilibrium (mg kg^−1^), k_1_ is the adsorption rate constant (h^−1^), and t is the adsorption time (h).

##### The Pseudo-Second-Order Kinetic Model

The pseudo-second-order kinetic model assumes that the adsorption rate is controlled by a chemisorption mechanism and that the adsorption capacity is proportional to the number of active sites on the adsorbent [20]. The equation is expressed as

(2) $$Q_{t}\;=\frac{k_{2}Q_{e}^{2}t}{1+k_{2}Q_{e}t}$$

where k_2_ is the pseudo-second-order kinetic equation adsorption rate constant (h^−1^).

##### The Elovich Model

The Elovich model is appropriate for describing the adsorption behavior on heterogeneous solid surfaces [21,22]. Recently, it has been applied to pollutant adsorption in aqueous solutions [23,24]. The equation is expressed as follows:

(3) $$Q_{t}\;=\;\frac{1}{K}\ln(1\;+\;\alpha \mathrm{Kt})$$

where α is the initial adsorption rate (mg kg^−1^ h^−1^), and K is the Elovich constant (kg mg^−1^).

##### Intraparticle Diffusion Model

The intraparticle diffusion model was used to fit the sorption kinetics to explain the mechanism in the sorption process [25], and can be described as follows:

(4) $$Q_{t}\;=\;Kt^{\frac{1}{2}}\;+\;C$$

where the K is the rate constant of stage (mg/(kg h^1/2^)). The C value can be obtained from the intercept of the stage and represents the constant depiction of resistance to mass transfer in the boundary layer.

#### 2.7.2. Adsorption Isotherm Models

##### Linear Model

The linear model is a common empirical model to describe the adsorption equilibrium of pollutants in a solid–liquid system. The equation is expressed as

(5) $$Q_{e}=aC_{e}+b$$

where Q_e_ is the amount of pollutant adsorbed at adsorption equilibrium (mg kg^−1^), C_e_ is the concentration of pollutant in solution at adsorption equilibrium (mg L^−1^), and a and b are constants.

##### Freundlich Modeling

Freundlich modeling is performed using an empirical equation that describes adsorption on heterogeneous surfaces, which assumes that the stronger binding sites are occupied first and that the binding strength decreases with increasing site occupancy [26]. The equation is expressed as

(6) $$Q_{e}=K_{F}C_{e}^{1/n}$$

where K_F_ is the Freundlich adsorption factor [(mg kg^−1^)/(mg L^−1^)^1/n^], representing the amount of pollutants adsorbed per unit of equilibrium concentration, and n is the degree of favorability of adsorption. A value of 1/n between 0.1 and 0.5 suggests favorable adsorption, while a value greater than 2 indicates that adsorption is difficult [27,28].

##### Langmuir Modeling

The Langmuir modeling describes the adsorption on a monolayer surface, assuming that all adsorption sites on the solid are identical and that there is no interaction between the adsorbed particles [29]. The equation is expressed as

(7) $$Q_{e}=Q_{\max}\frac{K_{L}C_{e}}{1+K_{L}C_{e}}$$

where Q_max_ is the maximum adsorption capacity of the adsorbent (mg kg^−1^) and K_L_ is the Langmuir equilibrium parameter (L mg^−1^).

#### 2.7.3. Soil Distribution Coefficient

Soil adsorption coefficient (K_d_) measures the amount of chemical substance adsorbed onto the soil per amount of water. Therefore, K_d_ was interpolated for each treatment and analyte for an aqueous concentration of 1.5 mg L^−1^, following Equations (8) and (9) [30]:

(8) $$K_{d}=\;\frac{Q_{e}}{C_{e}}=\;\frac{K_{F}C_{e}^{1/n}}{C_{e}}=\;K_{F}C_{e}^{\frac{1-n}{n}}$$

(9) $$K_{d}=\frac{Q_{e}}{C_{e}}=Q_{\max}\frac{K_{L}C_{e}}{1+K_{L}C_{e}}\frac{1}{C_{e}}=Q_{\max}\frac{K_{L}}{1+K_{L}C_{e}}$$

## 3. Results and Discussion

### 3.1. Physicochemical Properties of Soil

The physicochemical properties of the soil samples are shown in Table S1. The selected soils exhibited significant variations in their properties, making them representative of different soil types. The pH values of the soil samples ranged from 4.91 to 8.24, and the organic matter content varied between 3.31 and 54.71 g kg^−1^. The HLG, JX and GD samples had relatively high clay content (<0.002 mm), whereas other samples were predominantly composed of powdery particles (0.05–0.002 mm). The CEC of the soil samples ranged from 4.87 to 33.41 cmol(+) kg^−1^, with HLG samples having the highest CEC. Additionally, the soil samples were further analyzed for various forms of Fe and Mn, including amorphous Fe, amorphous Mn, free Fe, free Mn, total Fe, and total Mn. It was observed that Fe levels were generally higher than Mn across all samples. The total Fe content of the soil samples ranged from 17.22 to 37.40 g kg^−1^, while total Mn content ranged from 0.16 to 0.66 g kg^−1^. The free Fe content was consistently higher than amorphous Fe, while the differences between free and amorphous Mn content were minimal.

### 3.2. Adsorption Kinetics of TBBPA/S on Soil

**Figure 1:**
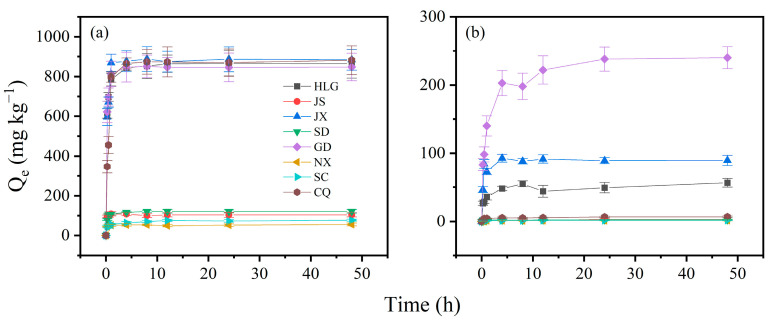
Adsorption kinetic curves of (**a**) TBBPA and (**b**) TBBPS in soil samples. Experimental conditions: soil = 0.5 g, [TBBPA] = 2 mg/L, [TBBPS] = 5 mg/L, water–soil = 20:1.

The adsorption variations in TBBPA and TBBPS on soil samples are shown in Figure 1. The adsorption capacities of TBBPA/S varied among different soils. For TBBPA, HLG, JX, GD, and CQ exhibited strong adsorption capacities, with adsorption amounts exceeding 800 mg kg^−1^. In contract, the adsorption of TBBPA on JS, SD, NX, and SC was below 150 mg kg^−1^, with NX adsorbing as little as 56 mg kg^−1^. For TBBPS, the highest adsorption was observed in GD (240 mg kg^−1^), followed by JX (89.6 mg kg^−1^) and HLG (56.4 mg kg^−1^), while the remaining soil samples adsorbed less than 10 mg kg^−1^. All soil samples adsorbed significantly more TBBPA than TBBPS, which was attributed to the higher octanol–water partition coefficient of TBBPA than that of TBBPS [31]. The higher partition coefficient allows TBBPA to adsorb more easily onto soil surfaces, while TBBPS is more soluble in water. The adsorption process of TBBPA/S on soil samples could be divided into two phases: a fast adsorption phase (0–4 h) and an equilibrium phase (4–48 h). In the fast adsorption phase, approximately 95% of the maximum adsorption occurred within 4 h. Adsorption gradually slowed after 4 h, entering the equilibrium phase, where approximately 5% of the total adsorption occurred. The fast phase of TBBPA lasted for a shorter time than that of TBBPS, suggesting that TBBPA adsorbed more quickly on soils, which is also attributed to its higher octanol–water partition coefficient. At the beginning of adsorption, TBBPA/S would quickly enter into SOM through partitioning [32] or quickly adsorb onto soil mineral surfaces via electrostatic adsorption [33]. Based on the kinetic analysis of TBBPA/S adsorption, 24 h was selected as the equilibrium time for adsorption experiments.

The adsorption kinetic data were fitted using the pseudo-first-order kinetic, pseudo-second-order kinetic, Elovich, and intraparticle diffusion models to evaluate the optimal kinetic parameters for predicting the adsorption process of TBBPA/S (Figures S2–S5). The simulation parameters and the correlation coefficients (R^2^) of these kinetic models for the adsorption of TBBPA and TBBPS are summarized in Table S2 and Table S3, respectively. As shown in Table S2, the R^2^ values of the four kinetic models were below 0.6 for JS and NX, indicating unsatisfactory fitting results. For the remaining six soils, the R^2^ ranges were 0.601–0.972 for the pseudo-first-order kinetic model, 0.808–0.999 for the pseudo-second-order kinetic model, and 0.621–0.910 for the Elovich model. As shown in Figure S2, the Q_e_ of TBBPA calculated from the pseudo-second-order kinetic model was closest to the Q_e_ obtained from the experiments. These results suggested that the adsorption process of TBBPA might be more consistent with the pseudo-second-order kinetic model. Intraparticle diffusion model was also used to analyze the adsorption kinetic data (Figure S3). As shown in Table S2, there were two linear regions observed in the intraparticle diffusion fitting plots for all eight soils: a linear region representing the surface loading at the beginning of the adsorption, and a second linear region representing the equilibrium stage. The results of two linear regions suggested that all soil samples have no more intraparticle diffusion during the adsorption process.

The modeling results for the adsorption kinetics of TBBPS are detailed in Table S3. The Elovich model yielded poor fits for JS, JX, SD, and NX, with R^2^ values below 0.6, indicating that these models were insufficient for describing TBBPS adsorption kinetics in these soils. In addition, the pseudo-first-order model for JS and CQ, and the pseudo-second-order model for SD and NX soils, also had R^2^ values lower than 0.6. Excluding soil samples with poor fits, the R^2^ ranges of the pseudo-first-order and the pseudo-second-order kinetic model for the remaining soil samples were 0.604–0.883 and 0.607–0.930, respectively, implying that the pseudo-second-order kinetic model provides a better description of TBBPS adsorption. As shown in Figure S4, the Q_e_ of TBBPS calculated from the pseudo-second-order kinetic model was closest to the Q_e_ obtained from the experiments. The analysis result of TBBPS data by the intraparticle diffusion model was similar to that of TBBPA. In summary, the adsorption kinetics of TBBPA/S varied across different soils, with the pseudo-second-order kinetic model being the most suitable.

### 3.3. Adsorption Isotherms of Soil for TBBPA/S

**Figure 2:**
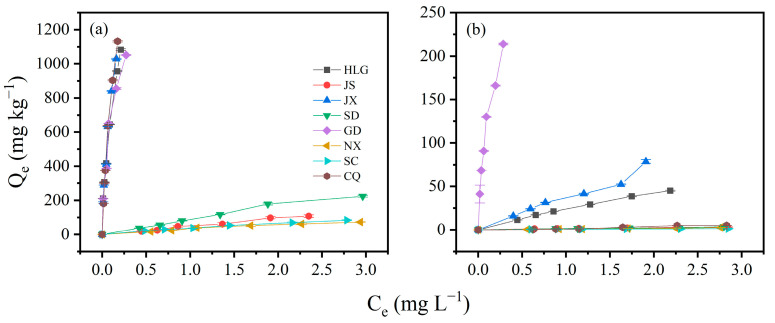
Adsorption isotherms of (**a**) TBBPA and (**b**) TBBPS on different soil samples.

The adsorption isotherms of TBBPA/S on different soil samples are shown in Figure 2. HLG, JX, GD, and CQ exhibited higher adsorption of TBBPA, while HLG, JX and GD showed higher adsorption of TBBPS, consistent with the adsorption kinetics results. The adsorption equilibrium data of TBBPA/S on different soils were fitted using the linear model, Freundlich model, and Langmuir model. The model parameters and R^2^ are provided in Tables S4 and S5. All three models adequately described the adsorption process of TBBPA on different soils, with R2 values exceeding 0.85. However, a comparison showed that the Linear model performed slightly worse for GD, with an R^2^ of 0.872, whereas both the Freundlich and Langmuir models had R^2^ values greater than 0.95, indicating a better fit. The R2 ranges for the Freundlich model and Langmuir model were 0.948–0.996 and 0.968–0.997, respectively, indicating that the Langmuir model provided the better fit. These results implied that the adsorption of TBBPA on soil was closer to monolayer adsorption. Generally, K_L_ in the Langmuir model represents the affinity between the adsorbate and the soil. The higher the K_L_ value, the greater the adsorption capacity. The order of K_L_ was as follows: GD > JX > HLG > CQ > NX > SD > SC > JS. Among the eight soils, HLG, CQ, GD, and JX had relatively strong adsorption capacities for TBBPA (Figure 2a), which was consistent with the K_L_ results.

The Langmuir model exhibited non-convergence when fitting the adsorption data of NX and CQ, and the R^2^ values of SD were below 0.8, indicating that the Langmuir model was unsuitable for describing the TBBPS adsorption on these soils. In contrast, both the Linear and Freundlich model could fit the TBBPS adsorption process well, with R^2^ exceeding 0.8. Further comparison revealed that Freundlich model performed slightly better than the Linear model, suggesting that the adsorption process of TBBPS on the soil conformed more closely to the Freundlich model, which assumed adsorption on the heterogeneous surface. This aligns with the complexity and heterogeneity of the soil samples. K_F_ represents the amount of pollutants adsorbed per unit of equilibrium concentration. The order of K_F_ was as follows: GD > JX > HLG > JS > SD > CQ > NX > SC. Among the eight soils, GD, JX, and HLG exhibited higher adsorption capacities for TBBPS (Figure 2b), consistent with the K_F_ results. n represents the degree of favorability of adsorption. The range of 1/n was 0.68–1.64, and only NX and CQ had 1/n values below 1, indicating that the adsorption of TBBPS involved multiple mechanisms [34].

The adsorption isotherms of TBBPA/S on different soils conformed to the Langmuir model and the Freundlich model, respectively, implying that the adsorption mechanisms of TBBPA and TBBPS might be different. The order of TBBPA adsorption on the eight soils was as follows CQ > JX > HLG > GD > SD > JS > SC > NX, and the order of TBBPS adsorption was GD > JX > HLG > CQ > SD ≈ JS ≈ NX ≈ SC. The differences in adsorption order further support the notion that the adsorption mechanism of TBBPA and TBBPS on soils may be governed by different processes. The adsorption of TBBPA on soil was closer to monolayer adsorption, while the adsorption of TBBPS tended to occur on heterogeneous surfaces.

TBBPA exhibited a pKa1 of 7.50, while TBBPS had a pKa1 of 4.74. Accordingly, in acidic soils (HLG, JX, GD, and CQ), TBBPA predominantly existed in molecular form, whereas TBBPS was present as an anion. As shown in Figure 2, the soil adsorption coefficient (Kd) values for TBBPA in acidic soils follow the order CQ > JX > HLG > GD, with their organic matter contents being 17.00, 10.11, 54.71, and 46.10 g/kg, respectively. This suggested that the adsorption of molecular TBBPA in acidic soils was not strongly correlated with soil organic matter (SOM) content. However, the similarity between the soil pH order (JX = GD < HLG < CQ) and the Kd order indicated that the adsorption of molecular TBBPA in acidic soils was closely related to soil ion type and ionic strength. In alkaline soils (SD, JS, SC, and NX), where TBBPA existed predominantly in ionic form, the Kd values follow the order SD > JS > SC > NX, while the soil pH order is JS < SC < SD < NX. This again suggested that adsorption of ionic TBBPA was governed by soil ion type and ionic strength. Given that TBBPS had a pKa1 of 4.74, it existed in anionic form across all soils. The Kd values for TBBPS followed the order GD > JX > HLG > CQ > JS > SD > NX > SC, while the soil pH values were ordered as JX = GD < HLG < CQ < JS < SC < SD < NX, which indicated that TBBPS adsorption was strongly influenced by soil ion type and ionic strength.

**Figure 3:**
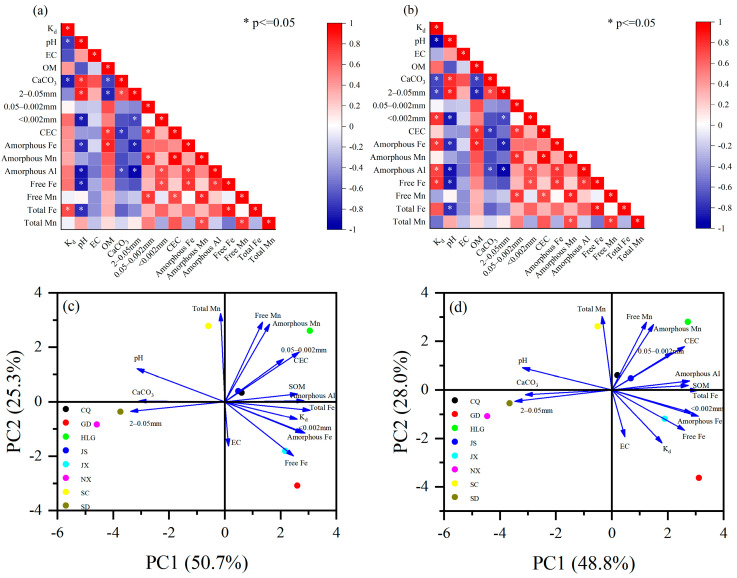
(**a**,**b**) Spearman correlation coefficients and (**c**,**d**) PCA between soil properties and TBBPA/S.

To further investigate the factors influencing the adsorption of TBBPA/S on soil, the correlation coefficients and PCA were analyzed between soil physicochemical properties and K_d_. The Shapiro–Wilk test was performed to assess the normality of K_d_ and soil physicochemical properties. The results showed that the K_d_ of TBBPA/S did not follow a normal distribution. Therefore, Spearman correlation coefficients were used for analysis. The correlation coefficients between the K_d_ of TBBPA/S and the soil properties are shown in Figure 3a,b, respectively, and the results of PCA are presented in Figure 3c,d. As shown in Figure 3a,b, the soil adsorption coefficient (K_d_) of TBBPA/S is positively correlated with soil organic matter, but the correlation is not significant. This indicates that other physicochemical properties of soil, such as pH, particle size distribution, and iron–manganese oxides, significantly influence TBBPA/S adsorption behavior.

For TBBPA, there was a negative correlation between K_d_ and pH (Figure 3a), which was consistent with the finding of Higgins et al. [35], who reported that adsorption of TBBPA decreased with increasing pH due to the effect of pH on TBBPA protonation. K_d_ was also negatively correlated with CaCO_3_ contents (Figure 3a), which was the one of the main causes of soil alkalinity [36]. Additionally, a significant positive correlation was found between K_d_ and total Fe content (Figure 3a). An increase in the content of iron oxides in soil leads to higher adsorption of pollutants [37], and ligand exchange and electrostatic interactions are the main mechanisms by which iron oxides adsorb pollutants [38,39]. As shown in Figure 3c, the K_d_ of TBBPA was strongly positive correlated with the total Fe, clay particles (<0.002 mm), amorphous Fe, and free Fe, while negative correlations were observed between the K_d_ of TBBPS and both pH and CaCO_3_ content.

For TBBPS, K_d_ showed a negative correlation with both pH, CaCO_3_ content, and sand content (2–0.05 mm) (Figure 3b). Similarly to TBBPA, the effect of pH and CaCO_3_ on the protonation of TBBPS resulted in a decrease in TBBPS adsorption as pH increased. The negative correlation between K_d_ and sand content indicated a positive correlation between K_d_ and particles smaller than 0.05 mm, implying that soils with a higher proportion of fine particles (<0.05 mm) exhibited better adsorption performance for TBBPS. The positive correlation between K_d_ and the clay particles (<0.002 mm) further confirmed that soils with higher clay mineral content exhibited the stronger adsorption of organic pollutants [40]. In addition, the positive correlation between K_d_ and the content of amorphous iron, amorphous aluminum, and free iron was observed (Figure 3b), suggesting that increasing the content of these components enhanced the soil’s adsorption capacity for TBBPS. These results were consistent with the results of PCA for TBBPS (Figure 3d). Amorphous Fe, a fine-grained iron mineral with a large specific surface area and high chemical activity, is often considered an adsorption site for pollutants. Amorphous Fe in soil showed a significant positive correlation with PFOS adsorption [41], and the K_F_ of PFOS was positively correlated with the content of Al_2_O_3_ and Fe_2_O_3_ in soil [42]. These results indicate that the effects of soil physicochemical properties on the adsorption coefficients of TBBPA and TBBPS differ, further supporting the conclusion that the adsorption mechanisms of TBBPA and TBBPS on soils are distinct. This is consistent with the adsorption isotherm model fitting results.

The differences in dissociation constants and octanol–water partition coefficients between TBBPA and TBBPS lead to certain differences in their performance on the same soil. For instance, HLG exhibited significantly higher adsorption of TBBPA than TBBPS (Figure 2). Due to the pH of HLG being 5.78, TBBPA remained in a molecular state, while TBBPS existed in an anionic form, which caused electrostatic repulsion between TBBPS and HLG, inhibiting its adsorption on HLG. In alkaline NX (pH = 8.89), both TBBPA and TBBPS existed in anionic form. The adsorption of TBBPA was also significantly higher than that of TBBPS (Figure 2), which may be attributed to its higher octanol–water partition coefficient.

### 3.4. Effect of pH on TBBPA/S Adsorption Isotherms

**Figure 4:**
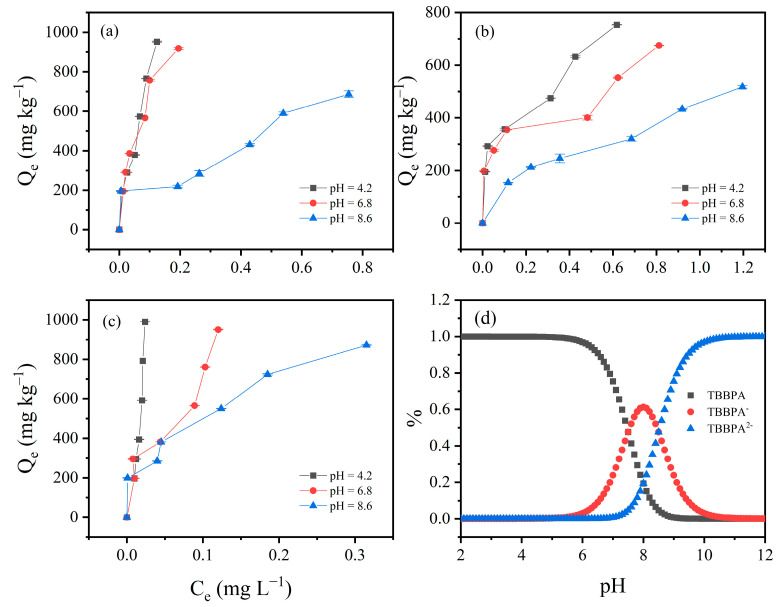
The effect of initial pH value on the adsorption of TBBPA by (**a**) HLG, (**b**) JX, and (**c**) GD. (**d**) The proportion of different components of TBBPA under different pH conditions.

**Figure 5:**
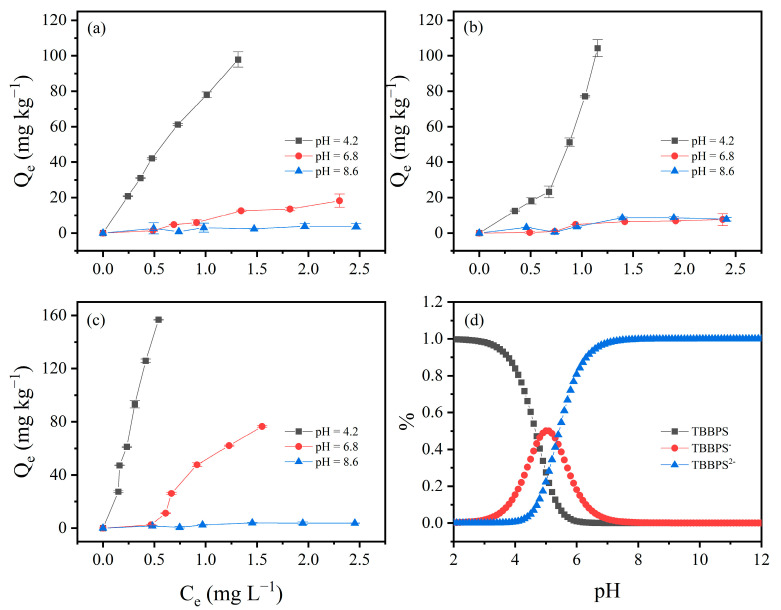
The effect of initial pH value on the adsorption of TBBPS by (**a**) HLG, (**b**) JX, and (**c**) GD. (**d**) The proportion of different components of TBBPS under different pH conditions.

Due to the high adsorption of HLG, JX, and GD for TBBPA/S, these three soils were selected to investigate the effect of pH on TBBPA/S adsorption isotherms. As shown in Figure 4, the adsorption isotherms of TBBPA for the three soil samples were examined at pH 4.2, 6.8 and 8.6. The adsorption of TBBPA by the three soil samples gradually decreased with the increasing pH, which may be related to changes in soil charge and the dissociation of TBBPA. With increasing pH, the soil surface carries more negative charge, and TBBPA transitions into its anionic forms due to its pKa_1_ and pKa_2_ (dissociation constant) values of 7.5 and 8.5, respectively. As shown in Figure 4d, neutral TBBPA dominated in the solution at pH = 4.2, while TBBPA^−^ and TBBPA^2−^ dominated at pH = 8.6, resulting in stronger electrostatic repulsion between TBBPA and the soil surface, thereby reducing adsorption [43]. A similar trend was observed for TBBPS (Figure 5), where adsorption by the soils gradually decreased with an increasing pH. Notably, the inhibitory effect of pH on the adsorption of TBBPA and TBBPS differs. At pH = 6.8, the adsorption of TBBPA by soil was slightly inhibited, but the adsorption of TBBPS by soil was significantly inhibited. This might be due to the different ionization behaviors of TBBPA and TBBPS (Figure 4d and Figure 5d). The adsorption of TBBPS by the soil was negligible at pH = 8.6, where TBBPS existed mainly in the form of TBBPA^2−^.

### 3.5. Effect of Soil Fractions on TBBPA/S Adsorption

**Figure 6:**
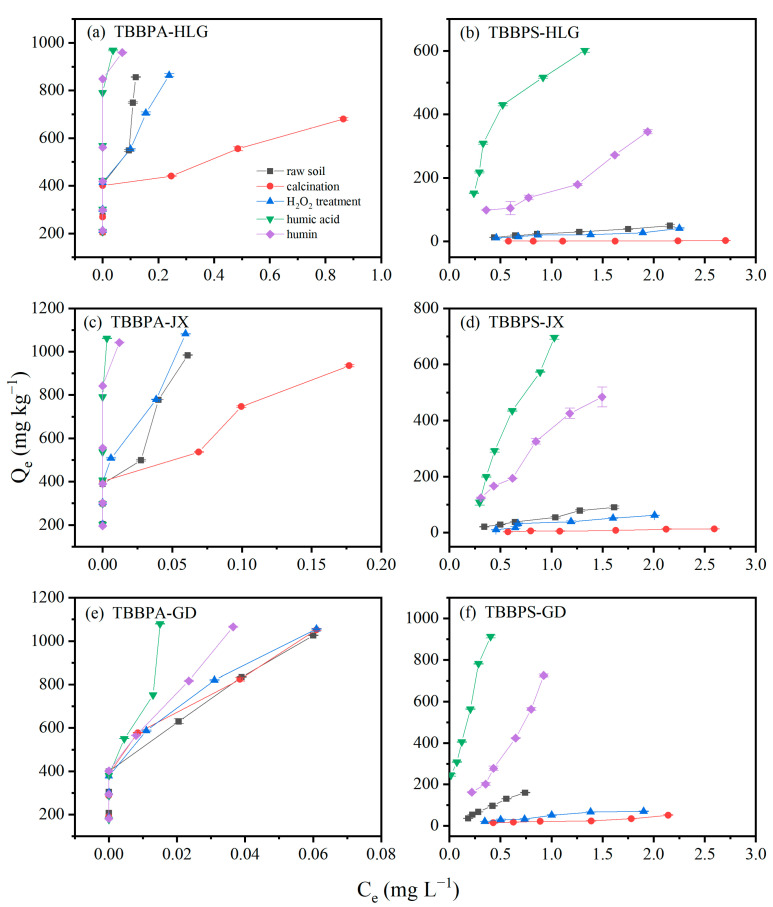
Isotherm of TBBPA adsorption by different components in (**a**) HLG, (**c**) JX and (**e**) GD. Isotherm of TBBPS adsorption by different components in (**b**) HLG, (**d**) JX and (**f**) GD.

The adsorption isotherms of TBBPA and TBBPS using different soil fractions are illustrated in Figure 6. As shown in Figure 6, the adsorption capacities of humin and humic acid from HLG, JX, and GD for TBBPA are significantly higher than those of TBBPS, which is attributed to the higher hydrophobicity of TBBPA compared to TBBPS. The distinct differences in the adsorption isotherms of TBBPA were observed for different soil fractions. For HLG and JX, the adsorption of TBBPA followed the order: humic acid ≈ humin > raw soil ≈ H_2_O_2_ treatment > calcination. However, the TBBPA adsorption in GD differed, with the order of humic acid > humin > raw soil ≈ H_2_O_2_ treatment ≈ calcination. Humic acid and humin are different components of SOM. Humic acid is organic matter that can dissolve in alkaline solution but not in acid solution. Humic acid is soluble in alkaline solutions but insoluble in acidic solutions, while humin is neither soluble in acid nor alkali and represents the primary form of organic matter in soils.

**Figure 7:**
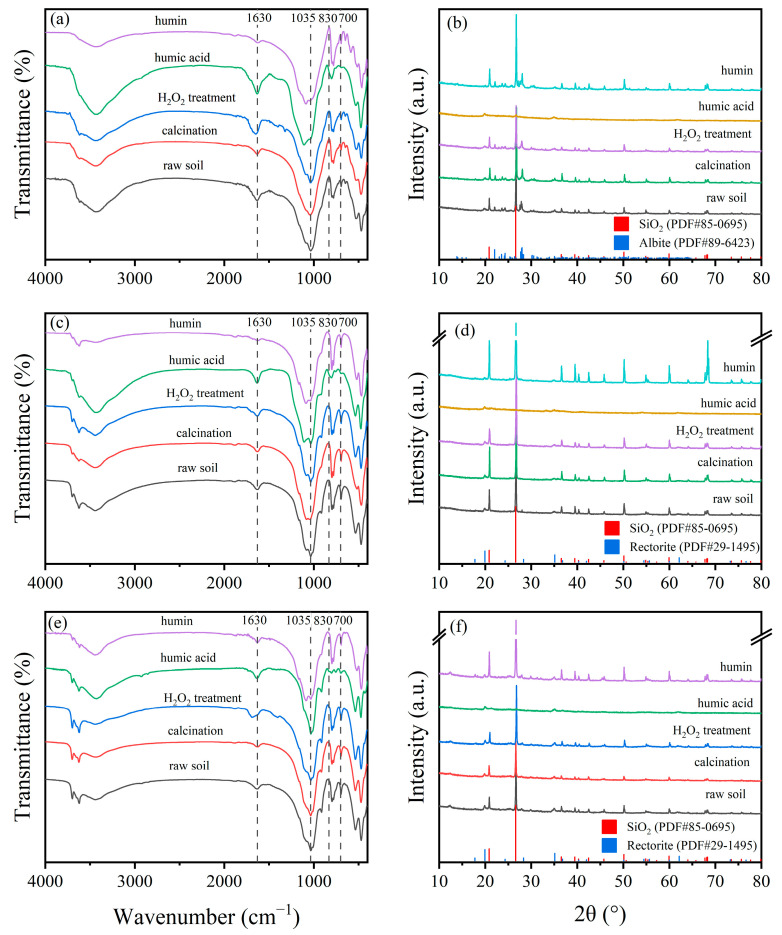
FTIR and XRD spectra of different components of (**a**,**b**) HLG, (**c**,**d**) JX and (**e**,**f**) GD.

The XRD patterns of different soil fractions are shown in Figure 7b,d,f. Diffraction peaks of SiO_2_ and Albite were detected in the raw soil of HLG. Except for humic acid, SiO_2_ and Albite were present in all other fractions of the HLG sample. Since the adsorption of TBBPA/S on humic acid was higher than that on raw soil, SiO_2_ and Albite were not the main adsorption sites in HLG. This was also observed in JX and GD, indicating that SiO_2_ and Rectorite were not the main adsorption sites in JX and GD. The FTIR spectra of different soil fractions are shown in Figure 7a,c,e. Compared with the raw soil, humic acid has higher absorbance at 1630 and 3440 cm^−1^, suggesting that a greater abundance of C=C and -OH functional groups. The absorbance between 830 and 700 cm^−1^ was significantly lower, suggesting that fewer benzene rings and condensed aromatic compounds were in humic acid. However, humin displayed lower absorbance at 1630 and 3440 cm^−1^ and higher absorbance at 830–700 cm^−1^, suggesting that humin had fewer C=C and -OH functional groups and more benzene rings and condensed aromatic structures. This composition contributed to higher surface hydrophobicity and stronger van der Waals forces during adsorption [44]. Therefore, the adsorption capacity of humic acid and humin for TBBPA was higher than that of the raw soil. H_2_O_2_ treatment and calcination are two methods used to remove soil organic matter to varying degrees. H_2_O_2_ treatment removed some reactive organic matter, while calcination eliminated most organic matter. Calcination significantly reduced the adsorption capacity of TBBPA in HLG and JX, which could be attributed to the removal of a large amount of organic matter. As shown in Figure 7a,c, the calcined soils had lower absorbance at 1630 and 3440 cm^−1^, indicating that organic matter containing C=C and -OH functional groups had been removed. However, calcination did not significantly affect the adsorption capacity of GD for TBBPA, likely due to the higher clay particle and Fe oxide content in GD (Table S1). Clay particles and Fe oxides are considered key adsorption sites for organic pollutants in soils [45]. No significant difference was observed in the adsorption capacity of the H_2_O_2_-treated samples compared to raw soil, which may be due to the limited removal of organic matter by H_2_O_2_. As shown in Figure 7, there was no significant difference in the FTIR spectra between raw soil and H_2_O_2_-treated soil. These findings suggest that the organic matter fraction, clay particles, and Fe oxide content were the main factors affecting TBBPA adsorption.

For TBBPS, the adsorption of different components of HLG, JX and GD followed the order: humic acid > humin > raw earth > H_2_O_2_ treatment > calcination. Unlike the adsorption behavior of TBBPA, humic acid exhibited a significantly higher adsorption capacity for TBBPS than humin, which may be due to TBBPS being more readily adsorbed onto organic matter containing C=C and -OH functional groups. The adsorption capacity of the H_2_O_2_-treated samples was slightly lower than that of the raw soil, potentially due to the oxidation of a portion of SOM. Calcined HLG, JX and GD showed almost no adsorption of TBBPS, indicating that SOM was the primary factor governing TBBPS adsorption. The adsorption of TBBPA/S by HLG, JX and GD was dominated by the organic matter fractions, clay particles, and the Fe oxide content.

### 3.6. Adsorption Mechanism of TBBPA/S by Soil

Based on the results of the pseudo-secondary kinetic model fitting for the adsorption kinetics of TBBPA/S, the adsorption process on soil was characterized as chemisorption. Furthermore, the results of correlation analysis, PCA, and the adsorption of different soil compositions revealed that the content of organic matter fractions, clay particles, and Fe oxides within the soil significantly influenced the adsorption of TBBPA/S. Soils with high organic matter and clay content are more likely to retain TBBPA [46]. In addition to hydrophobic and electrostatic interactions, hydrogen bonding also plays a role in the adsorption of TBBPA [47].

**Figure 8:**
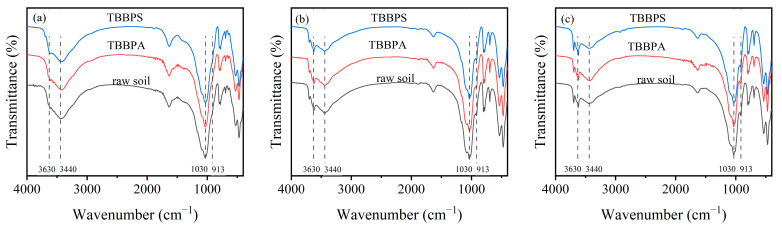
FTIR spectra of (**a**) HLG, (**b**) JX, and (**c**) GD before and after TBBPA/S adsorption.

To further explore the adsorption mechanism of TBBPA/S, FTIR spectra analysis was performed on soil samples before and after the adsorption of TBBPA/S. As shown in Figure 8, the bands at 1630 cm^−1^ in HLG, JX and GD correspond to the C=O vibration in carboxylate ions and amides, or C=C vibration in aromatics. The bands at 913, 1030, 3440, and 3630 cm^−1^ are attributed to the Al-OH bending vibrations, while the band at 795 cm^−1^ belongs to Fe-OH [48]. The bands at 533 and 473 cm^−1^ may be related to Fe-O vibrations. After the adsorption of TBBPA and TBBPS, the Al-OH vibrational band in HLG shifted from 3435 cm^−1^ to 3426 and 3424 cm^−1^, respectively, suggesting that hydrogen bonding may play a role in the adsorption of TBBPA/S in HLG [49]. Similarly, in GD, the Al-OH band shifted from 3441 cm^−1^ to 3430 cm^−1^ after TBBPA adsorption, while it remained at 3440 cm^−1^ after TBBPS adsorption, suggesting that the hydrogen bonding contributed to TBBPA adsorption but not to TBBPS. However, no changes in the FTIR spectra were observed for JX after the adsorption of TBBPA/S, suggesting that the adsorption process in JX was primarily driven by physical adsorption.

In conclusion, the adsorption kinetics of TBBPA/S on soils conform to the pseudo-second-order kinetic model. TBBPA adsorption follows the Langmuir model, whereas TBBPS adsorption aligns with the Freundlich model. Correlation analysis, PCA and adsorption experiments indicated that organic matter fractions, clay particles, and Fe oxides in the soil were critical factors influencing TBBPA/S adsorption. FTIR analysis further suggested that both physical adsorption and hydrogen bonding contribute to the adsorption mechanisms of TBBPA/S on soils. Based on these results, it is inferred that the adsorption of TBBPA on soil is mainly due to electrostatic adsorption, which is affected by soil pH and organic matter hydrogen bonds. However, the adsorption of TBBPS on soil is the result of multiple mechanisms, which are jointly affected by soil pH, minerals, and organic matter.

## 4. Conclusions

This study examines the adsorption characteristics of TBBPA and TBBPS across various Chinese soils. Adsorption kinetics followed pseudo-second-order models for both compounds. Isotherm analysis revealed distinct adsorption mechanisms: TBBPA adsorption was best described by the Langmuir model, while TBBPS adsorption aligned with the Freundlich model. These differences are primarily attributed to their contrasting octanol–water partition coefficients (K_ow_) and ionization behavior. Correlation analysis and PCA identified key soil properties influencing adsorption (K_d_). TBBPA adsorption showed strong negative correlations with soil pH and CaCO_3_ content, and a positive correlation with total Fe content. TBBPS adsorption exhibited strong negative correlations with soil pH, CaCO_3_ content, and sand content (2–0.05 mm), alongside positive correlations with clay content (<0.002 mm), amorphous Fe, amorphous Al, and free Fe content. Further analysis indicated soil organic matter (SOM) fractions significantly governed TBBPA/S adsorption. Collectively, the distinct adsorption behaviors of TBBPA/S on different soils provide fundamental data for predicting their environmental fate and elucidate the underlying mechanisms controlling their retention in soil systems. However, it should be noted that this study did not investigate competitive adsorption between TBBPA and TBBPS. Future research will focus on the potential effects of soil contamination age, competitive sorption, or the influence of microstructures and the physical state of organic matter.

## Data Availability

Data are available by contacting the authors.

## Supplementary Materials

The following supporting information can be downloaded at: https://www.mdpi.com/article/10.3390/toxics13080686/s1, Text S1: Determination and characterization of selected physicochemical properties of soil; Text S2: Analytical methods; Table S1: Physical and chemical properties of test soil; Table S2: The adsorption kinetics model of TBBPA on different soil samples; Table S3: The adsorption kinetics model of TBBPS on different soil samples; Table S4: Isothermal adsorption equation of TBBPA on soil samples; Table S5: Isothermal adsorption equation of TBBPS on soil samples; Figure S1. The molecule structure of TBBPA and TBBPS; Figure S2. Pseudo-first-order kinetic model, pseudo-second-order model and Elovich model for TBBPA adsorption kinetics to eight soils; Figure S3. Intraparticle diffusion model for TBBPA adsorption kinetics to eight soils; Figure S4. Pseudo-first-order kinetic model, pseudo-second-order model and Elovich model for TBBPS adsorption kinetics to eight soils; Figure S5. Intraparticle diffusion model for TBBPS adsorption kinetics to eight soils.

## Abbreviations

The following abbreviations are used in this manuscript:

TBBPA	Tetrabromobisphenol A
TBBPS	Tetrabromobisphenol S
PCA	Principal component analysis
BFRs	Brominated flame retardants
IARC	International Agency for Research on Cancer
SOM	Soil organic matter
CEC	Cation exchange capacity
IHSS	International Humic Substances Society
GPLC	High-performance liquid chromatography
