# Supplementary material for: Properties and Mechanisms of TBBPA and TBBPS Adsorption onto Various Soils in China
Source: Toxics. 2025 Aug 18;13(8):686. doi: 10.3390/toxics13080686 (PMC12389895; doi:10.3390/toxics13080686)
Supplement: Supplementary file 1 [file toxics-13-00686-s001.zip › toxics-3740143-supplementary.pdf]

Article

Properties and Mechanisms of TBBPA and TBBPS Adsorption onto Various Soils in China

Qi Wang [1,2], Aiguo Gu [1], Hongzhen Lian [2,*] and Jie Zou [1,*]

[1] *Jiangsu Product Quality Testing and Inspection Institute, Nanjing, Jiangsu 210007, China; qi.wang@mail.ecust.edu.cn*
[2] *State Key Laboratory of Analytical Chemistry for Life Science, School of Chemistry and Chemical Engineering, Nanjing University, Nanjing 210023, China*
* Correspondence: *hzlian@nju.edu.cn (Hongzhen Lian); 13951700473@139.com (Jie Zou)*

Text S1. Determination and characterization of selected physicochemical properties of soil

The physicochemical properties of the soils were determined using Chinese standard methods. Soil pH was measured following *soil –determination of pH –potentiometry* (HJ 962-2018), with a water – soil ratio of 1:2.5. Soil conductivity was assessed using *soil quality –determination of conductivity–electrode method* (HJ 802-2016), with a water–soil ratio of 1:5. The *soil–determination of organic carbon–potassium dichromate oxidation spectrophotometric method* (HJ 615-2011) was used to determine soil organic matter (SOM). *Soil quality–determination of cation exchange capacity (CEC)–Hexamminecobalt trichloride solution –spectrophotometric method* (HJ 889-2017) was used to measured cation exchange capacity (CEC). Calcium carbonate content was determined following the *determination of calcium carbonate in forest soil* (LY/T 1250-1999). *The determination of extracting iron, aluminum, manganese, silica, and carbon in forest soil* (LY/T 1257-1999) was used to determine the amorphous Fe, amorphous Mn, free Fe, and free Mn contents. Total Fe and Mn contents were analyzed by the *soil and sediment–determination of 11 elements–alkaline fusion and inductively coupled plasma optical emission spectrometry* (HJ 974-2018). Total Co and Cu contents were determined by *soil and sediment–determination of 19 total metal elements–inductively coupled plasma mass spectrometry* (HJ 1315-2023). *Soil–determination of particle size distribution–pipette method and hydrometer method* (HJ 1068-2019) was used to evaluated the particle size distribution. The crystal structure of clay-grained minerals was identified using X-ray diffraction spectrometry (XRD), and Fourier transform infrared spectroscopy (FTIR) was employed to analyze the functional group

structure of SOM.

Text S2. Analytical methods

Soil was mixed with methanol solution (methanol/water=1:1, v:v) with water–soil ratio of 10:1. The mixture was ultrasonicated at 25 °C for 30 min and centrifuged at 2100 × g for 5 min. Then, the supernatant was drawn through a 0.45 μm glass fiber, and the filtrate was placed in the liquid-phase vials. The concentration of TBBPA/S was quantified by a HPLC with a diode array detector (LC-20A, SHIMADZU), equipped with a C18 column (250 × 4.6 mm, 5 μm particle, Agilent). The mobile phases and detection wavelengths were set as follows: methanol and water (85:15 v/v) with $\lambda = 230$ nm for TBBPA at a flow rate of 1 ml min^{-1}; methanol and 1 mM ammonium acetate solution (30:70 v/v) with $\lambda = 224$ nm for TBBPS a flow rate of 1 ml min^{-1}.

Table S1 Physicochemical properties of test soil.

Tested soil	pH	Electrical conductivity (EC) (ms/cm)	Organic matter (g/kg)	$CaCO_3$ (g/kg)	Particle composition (%) 2 − 0.063mm (sand)	0.063 − 0.002mm (silt)	< 0.002mm (clayJ)	CEC (cmol/kg)	Amorphous Fe (g/kg)	Amorphous Mn (g/kg)	Amorphous Al (g/kg)	Free Fe (g/kg)	Free Mn (g/kg)	Total Fe (g/kg)	Total Mn (g/kg)
HLG	5.78	0.09	54.71	0.44	14.12	53.08	32.80	33.41	4.35	0.59	1.92	7.42	0.52	28.96	0.62
JS	7.44	0.12	22.56	5.14	21.76	69.16	9.08	18.43	5.06	0.22	0.87	13.06	0.23	32.73	0.36
JX	4.91	0.05	10.11	0.79	19.72	41.72	38.56	12.07	3.45	0.14	2.12	23.04	0.16	37.40	0.26
SD	8.24	0.13	8.04	45.55	62.84	31.82	5.34	5.40	0.35	0.09	0.29	3.18	0.04	18.12	0.41
GD	4.91	0.32	46.10	0.80	14.98	45.08	39.94	18.00	7.87	0.12	1.03	20.52	0.03	32.92	0.16
NX	8.89	0.15	3.31	118.16	64.51	22.98	12.52	4.87	0.21	0.07	0.21	4.11	0.05	17.22	0.34
SC	8.09	0.14	11.60	43.82	26.18	58.25	15.58	19.89	1.37	0.48	0.67	7.05	0.52	29.31	0.66
CQ	6.40	0.11	17.00	0.76	33.24	48.82	17.94	24.25	2.95	0.27	0.57	9.09	0.17	33.45	0.45

Table S2 The adsorption kinetics model of TBBPA on different soil samples.

Soil samples	Pseudo-primary kinetic modeling $Q_t = Q_e(1-e^{-kt})$	Pseudo-secondary kinetic modeling $Q_t=kQ_e^2t/(1+kQ_et)$	Elovich modeling $Q_t=\ln(1+aKt)/k$	Intraparticle diffusion model $Q_t=Kt^{1/2}+C$
HLG	$Qt = 851.67(1-e^{-4.40t})$<br>$R^2 = 0.834$	$Q_t=48676.05t/(1+56.25t)$<br>$R^2 = 0.999$	$Q_t=\ln(1+1.32\times10^8t)/0.025$<br>$R^2 = 0.768$	$Q_t=342.27t^{1/2}+448.44$, $t\leqslant1$<br>$R^2 = 0.979$<br>$Q_t=13.79t^{1/2}+807.13$, $1<t<48$<br>$R^2 =0.310$
JS	$Qt = 104.56(1-e^{-9.87t})$<br>$R^2 = 0.348$	$Q_t=6361.92t/(1+60.55t)$<br>$R^2 = 0.204$	$Q_t=\ln(1+1.07\times10^{43}t)/0.97$<br>$R^2 = -0.158$	$Q_t=25.46t^{1/2}+83.58$, $t\leqslant1$<br>$R^2 = 0.996$<br>$Q_t=-0.70t^{1/2}+107.46$, $1<t<48$<br>$R^2 =0.191$
JX	$Qt = 879.46(1-e^{-4.02t})$<br>$R^2 = 0.915$	$Q_t=7406.86t/(1+8.25t)$<br>$R^2 = 0.912$	$Q_t=\ln(1+1.25\times10^7t)/0.022$<br>$R^2 = 0.577$	$Q_t=557.30t^{1/2}+292.94$, $t\leqslant1$<br>$R^2 = 0.921$<br>$Q_t=2.50t^{1/2}+869.69$, $1<t<48$<br>$R^2 =0.401$
SD	$Qt = 120.32(1-e^{-4.29t})$<br>$R^2 = 0.908$	$Q_t=1058.44t/(1+8.62t)$<br>$R^2 = 0.948$	$Q_t=\ln(1+1.95\times10^7t)/0.16$<br>$R^2 = 0.690$	$Q_t=13.30t^{1/2}+90.18$, $t\leqslant8$<br>$R^2 = 0.979$<br>$Q_t=0.12t^{1/2}+121.23$, $8<t<48$<br>$R^2 =0.294$
GD	$Qt = 839.18(1-e^{-4.79t})$<br>$R^2 = 0.879$	$Q_t=9069.17t/(1+10.57t)$<br>$R^2 = 0.961$	$Q_t=\ln(1+7.94\times10^8t)/0.027$<br>$R^2 = 0.621$	$Q_t=383.31t^{1/2}+428.02$, $t\leqslant1$<br>$R^2 = 0.999$<br>$Q_t=3.95t^{1/2}+828.49$, $1<t<48$<br>$R^2 =0.312$
NX	$Qt = 53.37(1-e^{-6.98t})$<br>$R^2 = 0.582$	$Q_t=1237.70t/(1+22.93t)$<br>$R^2 = 0.478$	$Q_t=\ln(1+1.31\times10^{19}t)/0.87$<br>$R^2 = 0.296$	$Q_t=6.57t^{1/2}+43.18$, $t\leqslant1$<br>$R^2 = 0.463$<br>$Q_t=0.22t^{1/2}+52.32$, $1<t<48$<br>$R^2 =0.023$
SC	$Qt = 70.67(1-e^{-2.63t})$<br>$R^2 = 0.601$	$Q_t=316.44t/(1+4.31t)$<br>$R^2 = 0.808$	$Q_t=\ln(1+2371.99t)/0.14$<br>$R^2 = 0.910$	$Q_t=10.07t^{1/2}+42.13$, $t\leqslant12$

				$R^2 = 0.979$<br>$Q_t=0.53t^{1/2}+72.26$, $12<t<48$<br>$R^2=0.275$
CQ	$Qt = 875.61(1-e^{-1.76t})$<br>$R^2 = 0.972$	$Q_t=2436.31t/(1+2.70t)$<br>$R^2 = 0.934$	$Q_t=\ln(1+699.63t)/0.011$<br>$R^2 = 0.714$	$Q_t=296.15t^{1/2}+332.73$, $t \leqslant 4$<br>$R^2=0.655$<br>$Q_t=2.91t^{1/2}+860.21$, $4<t<48$<br>$R^2=0.732$

Table S3 The adsorption kinetics model of TBBPS on different soil samples.

Soil samples	Pseudo-primary kinetic modeling $Q_t = Q_e(1-e^{-kt})$	Pseudo-secondary kinetic modeling $Q_t=kQ_e^2t/(1+kQ_et)$	Elovich modeling $Q_t=ln(1+aKt)/k$	Intraparticle diffusion model $Q_t=Kt^{1/2}+C$
HLG	$Qt = 49.50(1-e^{-1.80t})$ $R^2 = 0.655$	$Q_t=155.90t/(1+3.06t)$ $R^2 = 0.758$	$Q_t=ln(1+919.28t)/0.19$ $R^2 = 0.783$	$Q_t=12.98t^{1/2}+20.31$, $t\leqslant 8$ $R^2 = 0.981$ $Q_t=0.48t^{1/2}+50.93$, $8<t<48$ $R^2 =0.040$
JS	$Qt = 2.18(1-e^{-2.39t})$ $R^2 = 0.302$	$Q_t=8.59t/(1+3.77t)$ $R^2 = 0.607$	$Q_t=ln(1+980.44t)/4.22$ $R^2 = 0.429$	$Q_t=2.14t^{1/2}-0.05$, $t\leqslant 1$ $R^2 = 0.933$ $Q_t=0.17t^{1/2}+1.49$, $1<t<48$ $R^2 =0.562$
JX	$Qt = 89.26(1-e^{-3.65t})$ $R^2 = 0.738$	$Q_t=623.43t/(1+6.79t)$ $R^2 = 0.697$	$Q_t=ln(1+1.57\times106t)/0.19$ $R^2 = 0.416$	$Q_t=26.26t^{1/2}+43.95$, $t\leqslant 1$ $R^2 = 0.630$ $Q_t=-0.36t^{1/2}+91.29$, $1<t<48$ $R^2 =0.102$
SD	$Qt = 2.31(1-e^{-2.89t})$ $R^2 = 0.604$	$Q_t=13.17t/(1+5.54t)$ $R^2 = 0.469$	$Q_t=ln(1+7.04\times10^5t)/6.99$ $R^2 = 0.292$	$Q_t=5.63t^{1/2}-2.15$, $t\leqslant 1$ $R^2 = 0.835$ $Q_t=0.073t^{1/2}+1.88$, $1<t<48$ $R^2 =0.272$
GD	$Qt = 219.69(1-e^{-1.14t})$ $R^2 = 0.883$	$Q_t=359.36t/(1+1.53t)$ $R^2 = 0.956$	$Q_t=ln(1+77.68t)/0.032$ $R^2 = 0.929$	$Q_t=82.77t^{1/2}+43.25$, $t\leqslant 4$ $R^2 = 0.971$ $Q_t=8.64t^{1/2}+185.19$, $4<t<48$ $R^2 =0.802$
NX	$Qt = 1.49(1-e^{-1.36t})$ $R^2 = 0.624$	$Q_t=2.95t/(1+1.90t)$ $R^2 = 0.589$	$Q_t=ln(1+223.47t)/5.61$ $R^2 = 0.505$	$Q_t=0.41t^{1/2}+0.39$, $t\leqslant 1$ $R^2 =0.057$ $Q_t=0.032t^{1/2}+1.30$, $1<t<48$ $R^2 =0.087$

SC	$Qt = 1.58(1-e^{-1.71t})$ $R^2 = 0.823$	$Q_t=4.16t/(1+2.51t)$ $R^2 = 0.930$	$Q_t=\ln(1+407.05t)/5.43$ $R^2 = 0.952$	$Q_t=0.39t^{1/2}+0.48$, $t \leqslant 8$ $R^2 = 0.979$ $Q_t=0.057t^{1/2}+1.37$, $8<t<48$ $R^2 =0.955$
CQ	$Qt = 5.83(1-e^{-1.98t})$ $R^2 = 0.584$	$Q_t=17.02t/(1+2.78t)$ $R^2 = 0.709$	$Q_t=\ln(1+459.48t)/1.49$ $R^2 = 0.851$	$Q_t=2.75t^{1/2}+0.82$, $t \leqslant 4$ $R^2 =0.687$ $Q_t=0.41t^{1/2}+4.04$, $4<t<48$ $R^2 =0.874$

Table S4 Isothermal adsorption equation of TBBPA on soil samples.

Soil samples	Linear modeling $Q_e = K_F C_e + b$	Freundlich modeling $Q_e = K_F C_e^n$	Langmuir modeling $Q_e = Q_{max}K_L C_e/(1+K_L C_e)$
HLG	$Q_e = 4062.53C_e+237.76$<br>$R^2 = 0.987$	$Q_e = 2465.62C_e^{0.55}$<br>$R^2 = 0.99$<br>$K_F = 2465.62$<br>$1/n = 1.82$	$Q_e = 11962.21C_e/(1+6.46C_e)$<br>$R^2 = 0.990$<br>$Q_{max} = 1851.74$<br>$K_L = 6.46$
JS	$Q_e = 47.50C_e-0.68$<br>$R^2 = 0.969$	$Q_e = 45.58C_e^{1.09}$<br>$R^2 = 0.958$<br>$K_F = 45.58$<br>$1/n = 0.92$	$Q_e = 47.98C_e/(1+0.009C_e)$<br>$R^2 = 0.968$<br>$Q_{max} = 5331.11$<br>$K_L = 0.009$
JX	$Q_e = 5209.77C_e+218.55$<br>$R^2 = 0.959$	$Q_e = 3269.72C_e^{0.63}$<br>$R^2 = 0.982$<br>$K_F = 3269.72$<br>$1/n = 1.59$	$Q_e = 13324.52C_e/(1+6.74C_e)$<br>$R^2 = 0.986$<br>$Q_{max} = 1976.93$<br>$K_L = 6.74$
SD	$Q_e = 72.27C_e+21.96$<br>$R^2 = 0.933$	$Q_e = 98.44C_e^{0.79}$<br>$R^2 = 0.951$<br>$K_F = 98.44$<br>$1/n = 1.27$	$Q_e = 117.09C_e/(1+0.17C_e)$<br>$R^2 = 0.968$<br>$Q_{max} = 688.76$<br>$K_L = 0.17$
GD	$Q_e = 2631.44C_e+384.62$<br>$R^2 = 0.872$	$Q_e = 1964.10C_e^{0.47}$<br>$R^2 = 0.953$<br>$K_F = 1964.10$<br>$1/n = 2.13$	$Q_e = 12778.12C_e/(1+8.60C_e)$<br>$R^2 = 0.970$<br>$Q_{max} = 1485.83$<br>$K_L = 8.60$
NX	$Q_e = 22.80C_e+10.04$<br>$R^2 = 0.937$	$Q_e = 33.81C_e^{0.74}$<br>$R^2 = 0.962$<br>$K_F = 33.81$<br>$1/n = 1.28$	$Q_e = 41.88C_e/(1+0.23C_e)$<br>$R^2 = 0.980$<br>$Q_{max} = 182.09$<br>$K_L = 0.23$
SC	$Q_e = 27.16C_e+10.02$<br>$R^2 = 0.991$	$Q_e = 37.88C_e^{0.78}$<br>$R^2 = 0.996$<br>$K_F = 37.88$<br>$1/n = 1.28$	$Q_e = 44.24C_e/(1+0.17C_e)$<br>$R^2 = 0.997$<br>$Q_{max} = 260.25$<br>$K_L = 0.17$
CQ	$Q_e = 4913.85C_e+315.44$<br>$R^2 = 0.882$	$Q_e = 2982.62C_e^{0.54}$<br>$R^2 = 0.948$<br>$K_F = 2982.62$<br>$1/n = 1.85$	$Q_e = 13168.16C_e/(1+6.01C_e)$<br>$R^2 = 0.996$<br>$Q_{max} = 2191.04$<br>$K_L = 6.01$

Table S5 Isothermal adsorption equation of TBBPS on soil samples.

Soil samples	Linear modeling $Q_e = K_F C_e + b$	Freundlich modeling $Q_e = K_F C_e^n$	Langmuir modeling $Q_e = Q_{max} K_L C_e/(1+K_L C_e)$
HLG	$Q_e = 18.85C_e+4.59$<br>$R^2 = 0.993$	$Q_e = 24.00C_e^{0.81}$<br>$R^2 = 0.997$<br>$K_F = 24.00$<br>$1/n = 1.23$	$Q_e = 28.57C_e/(1+0.18C_e)$<br>$R^2 = 0.999$<br>$Q_{max} = 158.72$<br>$K_L = 0.18$
JS	$Q_e = 0.98C_e-0.70$<br>$R^2 = 0.971$	$Q_e = 1.59C_e^{0.67}$<br>$R^2 = 0.973$<br>$K_F = 1.59$<br>$1/n =1.49$	$Q_e = 2.03C_e/(1+0.29C_e)$<br>$R^2 = 0.966$<br>$Q_{max} = 7$<br>$K_L =0.29$
JX	$Q_e = 28.16C_e+7.22$<br>$R^2 = 0.979$	$Q_e = 36.08C_e^{0.77}$<br>$R^2 = 0.989$<br>$K_F = 36.08$<br>$1/n =1.30$	$Q_e = 48.19C_e/(1+0.31C_e)$<br>$R^2 = 0.993$<br>$Q_{max} = 155.45$<br>$K_L =0.31$
SD	$Q_e = 1.00C_e+0.36$<br>$R^2 = 0.828$	$Q_e = 1.34C_e^{0.83}$<br>$R^2 = 0.814$<br>$K_F = 1.34$<br>$1/n =1.20$	$Q_e =1.39C_e/(1+0.082C_e)$<br>$R^2 = 0.798$<br>$Q_{max} = 16.95$<br>$K_L =0.082$
GD	$Q_e = 672.11C_e+30.24$<br>$R^2 = 0.966$	$Q_e = 459.31C_e^{0.61}$<br>$R^2 = 0.991$<br>$K_F = 459.31$<br>$1/n =1.64$	$Q_e = 2348.70C_e/(1+7.90C_e)$<br>$R^2 = 0.990$<br>$Q_{max} = 297.30$<br>$K_L =7.90$
NX	$Q_e = 0.93C_e-0.19$<br>$R^2 = 0.977$	$Q_e = 0.73C_e^{1.19}$<br>$R^2 = 0.982$<br>$K_F = 0.73$<br>$1/n =0.84$	N.C.
SC	$Q_e = 0.39C_e+0.18$<br>$R^2 = 0.984$	$Q_e = 0.59C_e^{0.73}$<br>$R^2 = 0.988$<br>$K_F = 0.59$<br>$1/n =1.37$	$Q_e = 0.75C_e/(1+0.25C_e)$<br>$R^2 = 0.985$<br>$Q_{max} = 3$<br>$K_L =0.25$
CQ	$Q_e = 2.55C_e-1.44$<br>$R^2 = 0.941$	$Q_e = 1.27C_e^{1.46}$<br>$R^2 = 0.909$<br>$K_F = 1.27$<br>$1/n =0.68$	N.C.

N.C.：non-convergence

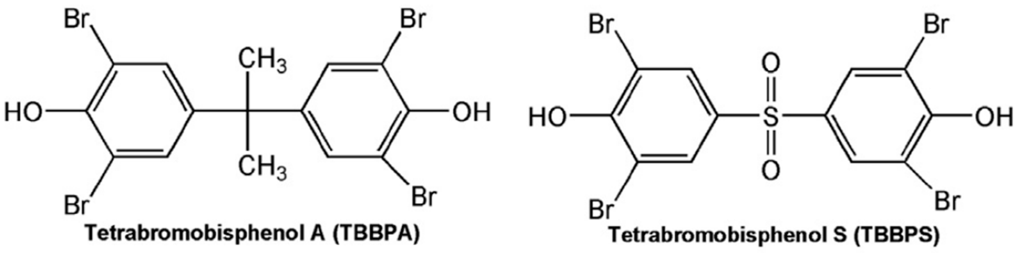

Figure S1. The molecule structure of TBBPA and TBBPS.

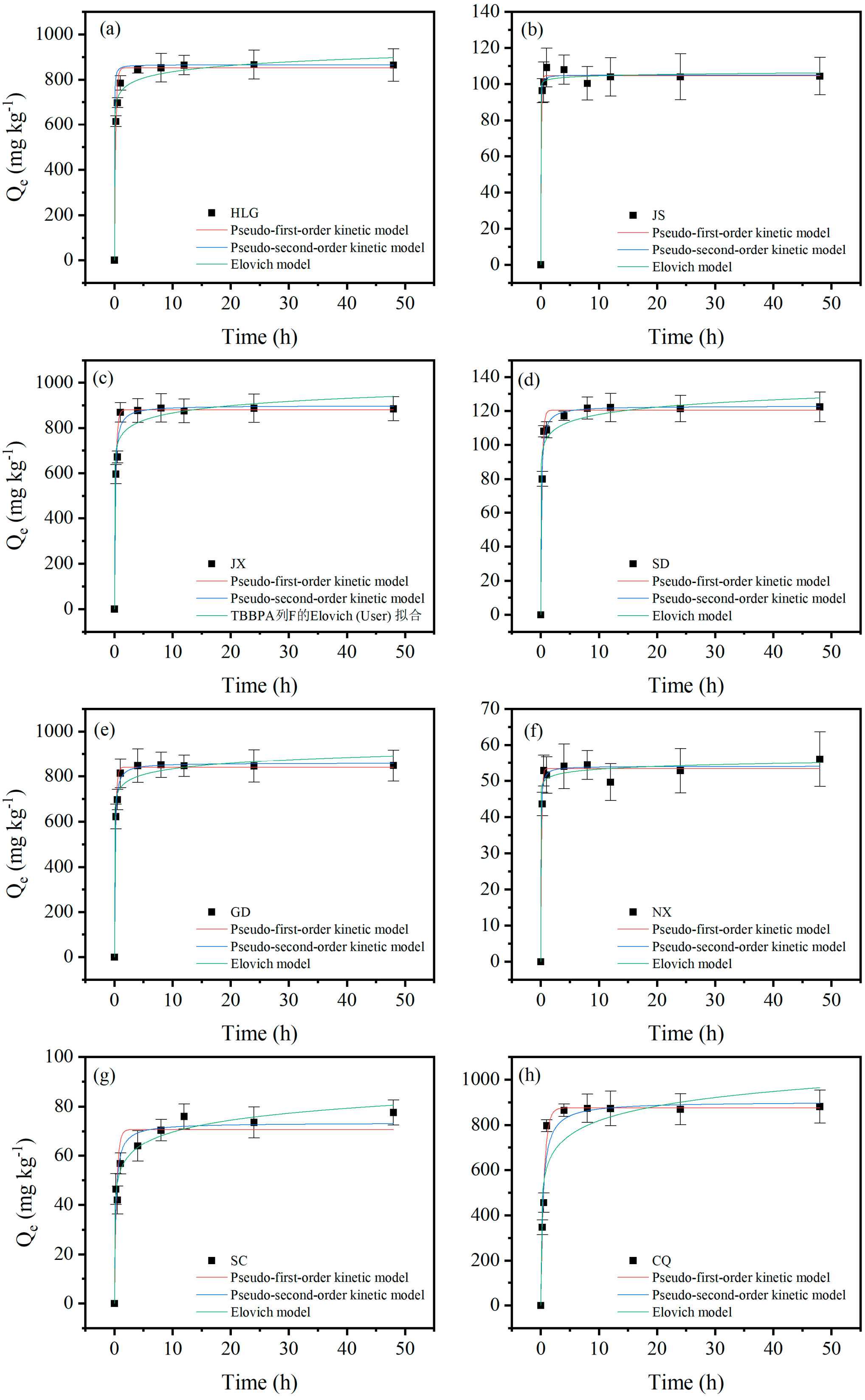

Figure S2. Pseudo-first-order kinetic model, pseudo-second-order model, and Elovich model for TBBPA adsorption kinetics to eight soils.

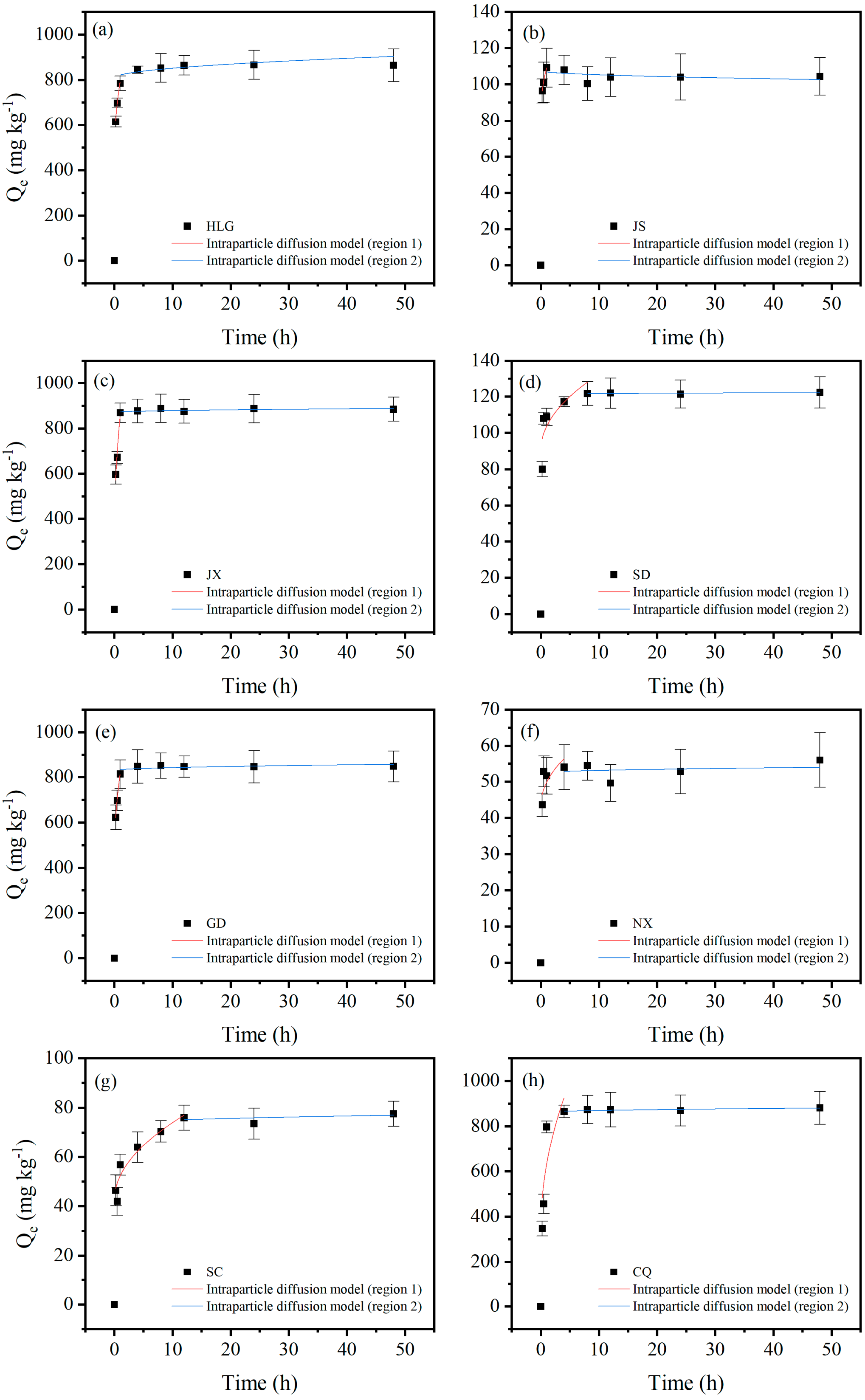

Figure S3. Intraparticle diffusion model for TBBPA adsorption kinetics to eight soils.

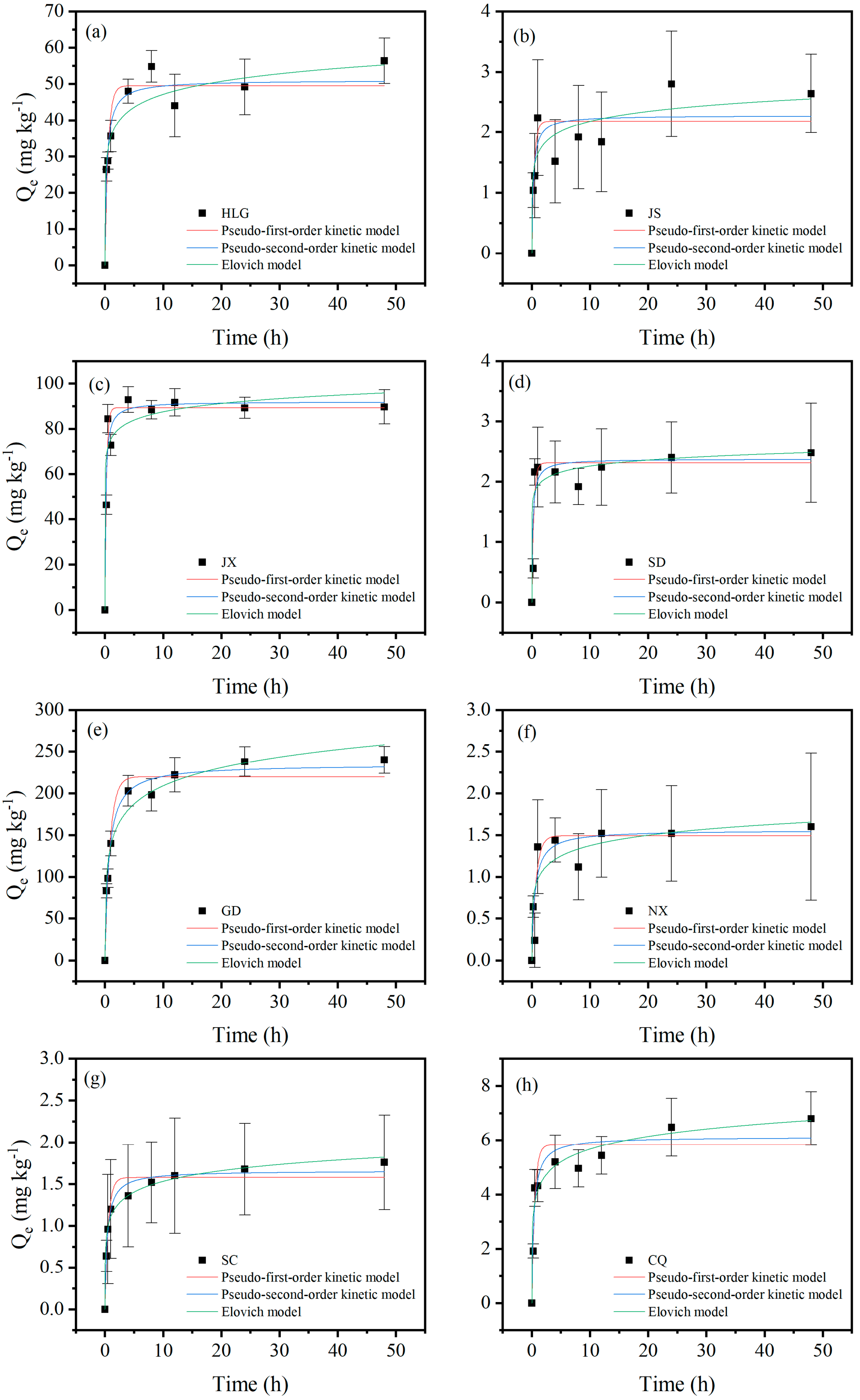

Figure S4. Pseudo-first-order kinetic model, pseudo-second-order model, and Elovich model for TBBPS adsorption kinetics to eight soils.

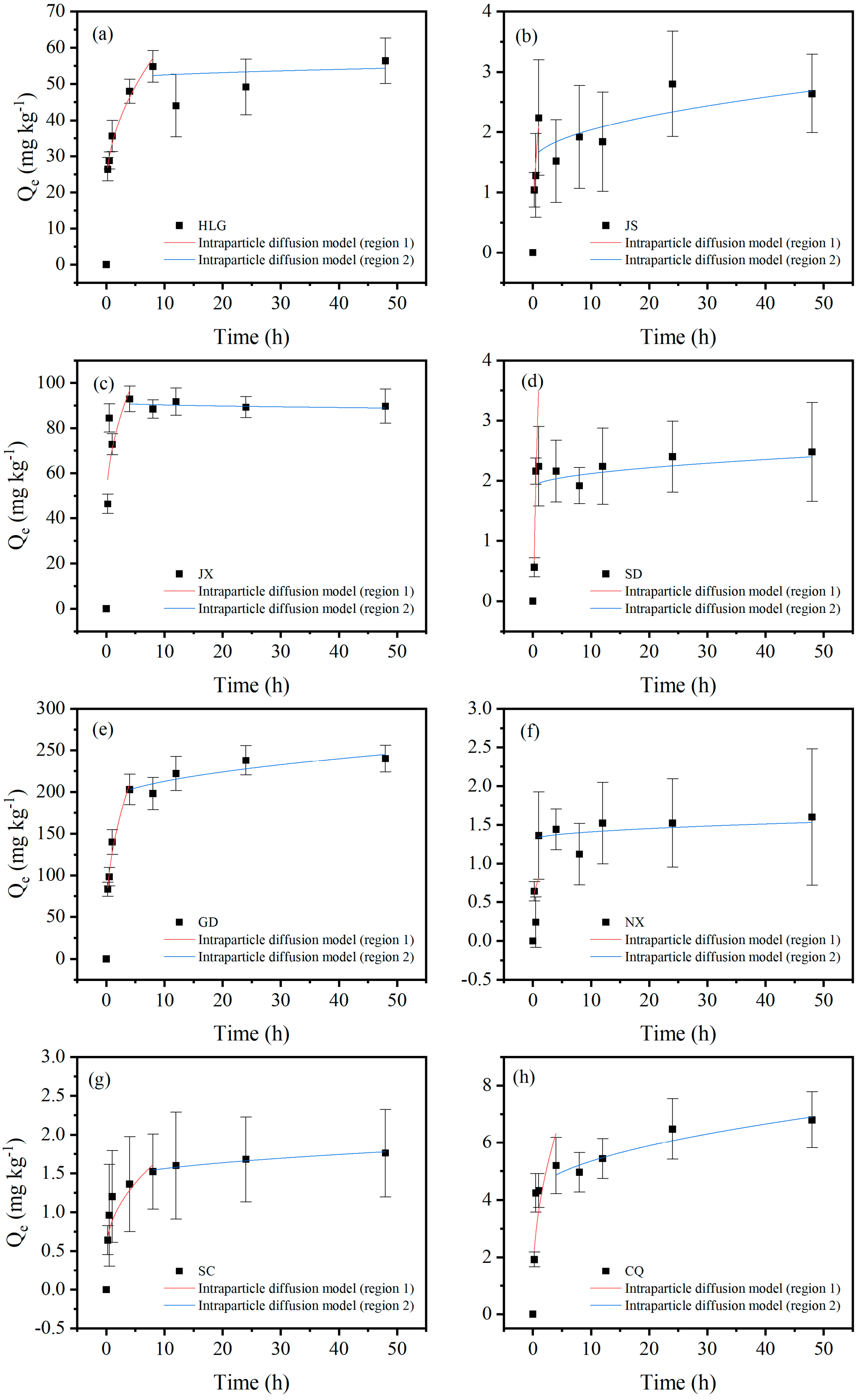

Figure S5. Intraparticle diffusion model for TBBPS adsorption kinetics to eight soils.
